# Quantitative evaluation of microvacuole formation in five intraocular lens models made of different hydrophobic materials

**DOI:** 10.1371/journal.pone.0250860

**Published:** 2021-04-30

**Authors:** Timur M. Yildirim, Sonja K. Schickhardt, Qiang Wang, Elfriede Friedmann, Ramin Khoramnia, Gerd U. Auffarth

**Affiliations:** 1 The David J. Apple International Laboratory for Ocular Pathology, Department of Ophthalmology, University of Heidelberg, Heidelberg, Germany; 2 Department of Ophthalmology, The Third Affiliated Hospital of Wenzhou Medical University, Rui’an, Zhejiang, China; 3 Faculty of Mathematics and Natural Sciences, University of Kassel, Kassel, Germany; Aston University School of Life and Health Sciences, UNITED KINGDOM

## Abstract

In this laboratory study, we assessed the resistance to microvacuole (glistening) formation in hydrophobic intraocular lenses (IOLs). Glistenings were induced in five lenses each of five different hydrophobic acrylic IOL models, using an established in vitro laboratory model: 800C (Rayner, Worthing, UK), AcrySof SN60WF (Alcon, Fort Worth, USA), Tecnis ZCB00 (Johnson & Johnson Vision, Santa Ana, USA), Vivinex XY1 (Hoya, Tokyo, Japan) and CT Lucia 611P (Zeiss, Oberkochen, Germany). We evaluated the number of microvacuoles per square millimeter (MV/mm^2^) in the central part of each IOL. Results were analyzed statistically, and mean glistening numbers were ranked, with the highest in the SN60WF which had 66.0 (±45.5) MVs/mm, followed by the 611P with 30.7 (±8.4) MVs/mm^2^. The 800C and XY1 showed comparable values of 2.0 (±3.6) and 2.7 (±2.4) MVs/mm^2^, respectively. ZCB00 had the lowest number with 0.9 (±0.6) MVs/mm^2^. This study shows that the resistance to glistening formation differs depending on the hydrophobic acrylic copolymer composition of the IOL material. Some IOLs from current clinical use are still prone to develop glistenings whereas others, including the ZCB00, 800C and XY1 show high resistance to microvacuole formation.

## Introduction

Since the 1990s, intraocular lenses (IOLs) made of hydrophobic acrylic copolymers have become increasingly popular. However, after implantation, the IOL material can alter, and over time this change can decrease the implant’s optical clarity [1–3]. On slit-lamp examination, these changes have been described as a whitish opacity or glittering inclusions. They were first described in 1984 and are now widely referred to as glistenings [4]. The most accepted theory regarding glistenings’ formation is that water entering the material collects in low polymer density areas (pockets). As the pockets enlarge over time, vacuoles might form which are visible to the clinician at the slit-lamp [5, 6]. Glistenings are 1 to 25 μm in size; vacuoles of less than 200 nm in diameter distributed up to 120 μm underneath the IOL surface are termed subsurface nanoglistenings (SSNGs) [7].

Depending on the number of vacuoles seen in slit-lamp examination, clinical grading scales are used to categorize glistening severity [8, 9]. As the formation of glistenings usually takes months to years in-vivo, laboratory methods for accelerated aging have been developed to simulate this condition [2]. In accordance with the clinical grading system, IOLs can then be divided into different glistening categories depending on the number of microvacuoles per square millimetre that are produced after the aging procedure [6]. Such methods have been used to study the impact of glistenings on the optical performance: whereas the effect of glistenings on the visual acuity seems to be rather small, their impact on intraocular light scattering is higher and shows a positive linear correlation with an increasing number of vacuoles [10, 11].

A popular hydrophobic IOL material is the AcrySof (Alcon, Fort Worth, USA). The material is known for its tendency to develop glistenings, resulting from the biomaterial’s properties and the IOL manufacture. The hygroscopic nature of a polymer changes depending on the temperature and ionic strength of the surrounding solution [12]. As water diffuses into the polymer due to equilibrium driving forces, discreetly visible vacuoles can develop [5]. However, why some hydrophobic IOLs such as the AcrySof, are more prone to form glistenings than others, is poorly disclosed in the literature [2]. Reference has been made to unspecified improvements in the AcrySof manufacturing that have decreased the tendency over time [13, 14]. In more recently introduced hydrophobic IOL materials, the manufacturer has modified the copolymer’s chemical content, or the process of polymerisation or the IOL production to reduce the propensity for glistening formation. The clinical follow-up period for some of these IOLs still is too short to conclude on their tendency to form in-vivo glistenings. In our study, we induced glistenings in different hydrophobic acrylic IOLs that are in current clinical use. We subjected them to an accelerated aging procedure in the laboratory where our aim was to evaluate in-vitro glistening numbers and compare the in-vitro tendency to form glistenings.

## Methods

### Intraocular lenses

Five different models of monofocal IOL were chosen that have a single-piece design that can be implanted through a 2.2 mm incision. Five lenses from each model were tested—all having the same refractive power of +21.0 diopters. Table 1 summaries the specifications of the lens models.

**Table 1:** Characteristics of the studied intraocular lenses.

IOL model (manufacturing date)	Manufacturer	Material Trademark	Optic Copolymer*	Equilibrium Water Content (in percent)	Blue-Light Filter	Refractive index	Manufacturing process
800C (2017)	Rayner	n.a.	Cross-linked hydrophobic polyurethane acrylates and methacrylates	< 3.0	No	1.51	Cast-moulding
SN60WF (2017)	Alcon	AcrySof IQ	Phenylethyl acrylate (PEA) and phenylethyl methacrylate (PEMA) cross-linked with butanediol diacrylate (BDDA)	0.1–0.5	Yes	1.55	Cast-moulding
ZCB00 (2016)	Johnson& Johnson Vision	Tecnis	Ethyl acrylate, ethyl methacrylate, 2,2,2-trifluoroethyl methacrylate cross-linked with ethyl glycol dimethacrylate	< 1.0	No	1.47	Lathe-cut
XY1 (2016)	Hoya	Vivinex	Cross-linked phenylethyl methacrylate (PEMA) and *n*-butyl acrylate, fluoroalkyl methacrylate	< 1.0	Yes	1.52	Cast-moulding
CT Lucia 611P (2016)	Carl Zeiss Meditec	n.a.	Cross-linked butyl acrylate, ethyl methacrylate and N-benzyl-N-isopropylpropenamide, heparin-coated surface	0.3	No	1.49	Lathe-cut

IOL intraocular lens; n.a. not applicable;

*Information on Optic Copolymer was provided by the manufacturer.

### In vitro glistening formation

Microvacuoles (glistenings) were induced in vitro by temperature changes using an established accelerated aging protocol which was described in detail in earlier publications [6, 10, 11]. In short, the lenses were hydrated in Sodium Chloride solution (0.9%) in glass flasks and stored in an oven at 45°C for 24 hours. After removal from the oven, the temperature was reduced to 37°C by immersing the flasks in a water-bath for 2.5 hours. Imaging was performed immediately thereafter at a temperature of 37°C.

### Evaluation of glistenings

**Fig 1:**
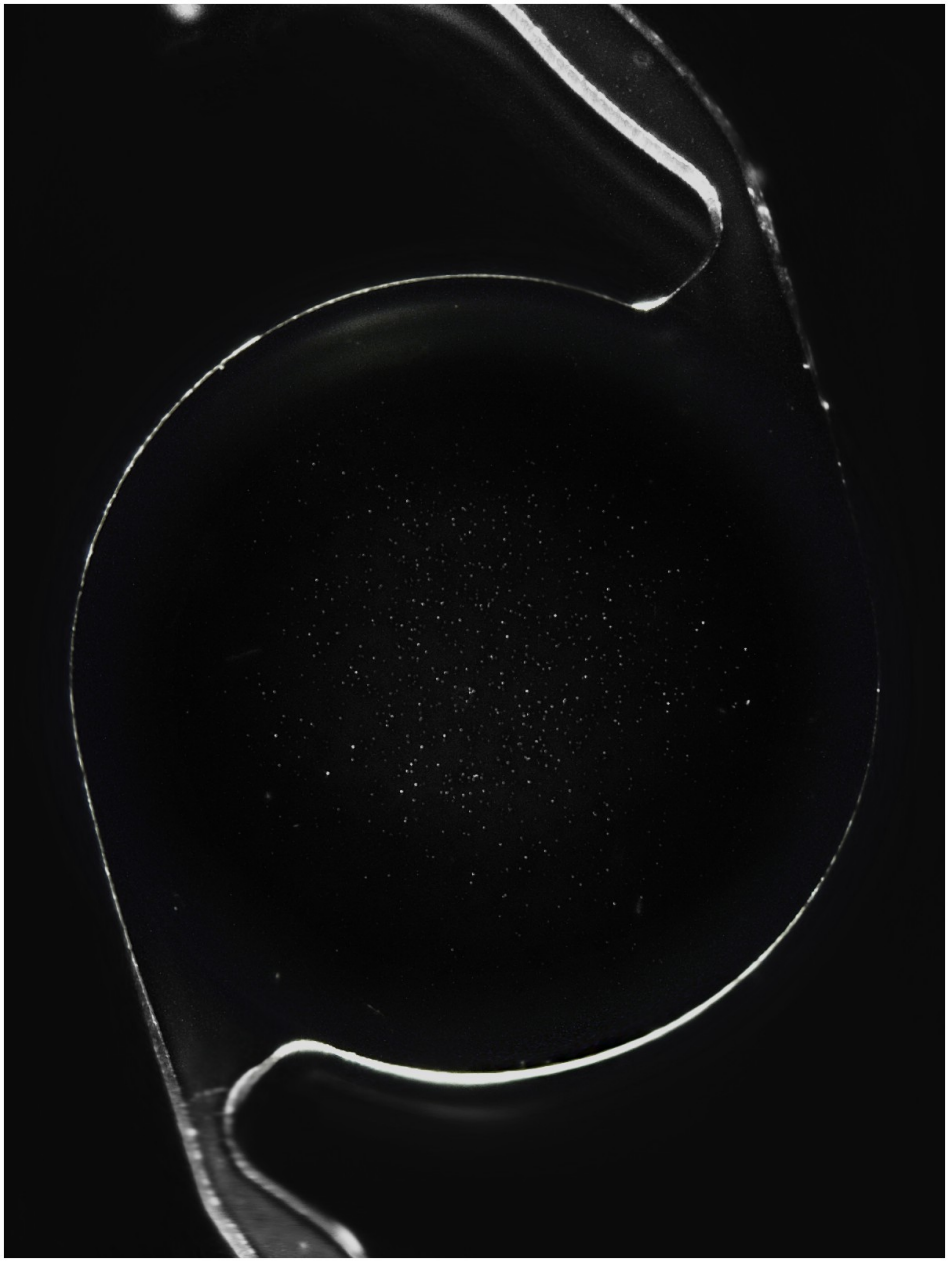
Overview image in 14x magnification of one of the SN60WF lenses after glistening induction. Glistenings are most numerous in the central part of the IOL optic.

The lenses were examined under an EMZ-8TR Trinocular Zoom Stereo microscope (Meiji Techno, Saitama, Japan). Each lens was moved into a Sodium Chloride solution (0.9%) on top of a heated stage to maintain the aqueous surrounding and the temperature of the IOL at 37°C during imaging to preserve MV density and size during image acquisition. Microscopic images of all IOLs were taken immediately after the aging process using an Infinity-2CB digital camera (Lumenera, Nepean, Canada). After obtaining one overview image in 14x magnification of each IOL’s whole optic (Fig 1), the lens was centred and magnification was turned to 90x to obtain an image of the central part of the optic.

The central section was selected to correspond to the region with the highest glistening numbers. After all photographs were obtained, image analysis was performed using the ImageJ software 1.49v using the same settings for each IOL as published in previous studies [6]. A single investigator (QW) performed image analysis who was blinded to minimize subjective influence. Before analysis, test samples with low to high glistening numbers have been used to predefine all parameters. Analysis was subsequently performed using the same predefined settings for all images. To account for slight fluctuation in image illumination, first we manually adjusted brightness and contrast up to the point where the background of each image appeared uniform. Further image analysis was performed using a semi-automated approach: for every image, the algorithm *Color Threshold* with the *Intermodes* method was applied to dichotomize the image information. After that *Analyze Particles* was used with the size of particles set from 25 to infinity to account for background noise. By this we obtained the glistening count in each 1200 x 1600 pixels image. An image of a micrometer in 90x magnification was used to calibrate results to determine the density of glistenings per square millimetre. As 1 mm corresponded to 1086 Pixels and the original image size was 1200 Pixels x 1600 Pixels, total image size was 1.63 mm^2^. The given number of glistenings was divided by 1.63 to obtain the number of microvacuoles per square millimetre (MVs/mm^2^) [10, 11]. We compared the number of glistenings to a grading scale, which has been modified from the well-known Miyata scale, as in our previous studies [6, 8, 11]. Results were grouped in the following grades to compare the laboratory measurements to a clinical impact: Grade 0 (< 25 MVs/mm^2^), grade 1 (25–100 MVs/mm^2^), grade 2 (100–200 MVs/mm^2^), grade 3 (> 200 MVs/mm^2^).

### Data analysis

Statistical analysis was performed with SPSS (IBM SPSS Statistics, V.22). Descriptive statistics included mean number of MVs/mm^2^ (±standard deviation) for five IOLs from each model. The QQ plot revealed that data did not satisfy normality distribution, so analysis was performed using Kruskal-Wallis test. Mann-Whitney-U nonparametric test with Bonferroni correction was used for post-hoc comparison. A P-value less than 0.05 was considered statistically significant.

## Results

### Material quality

**Fig 2:**
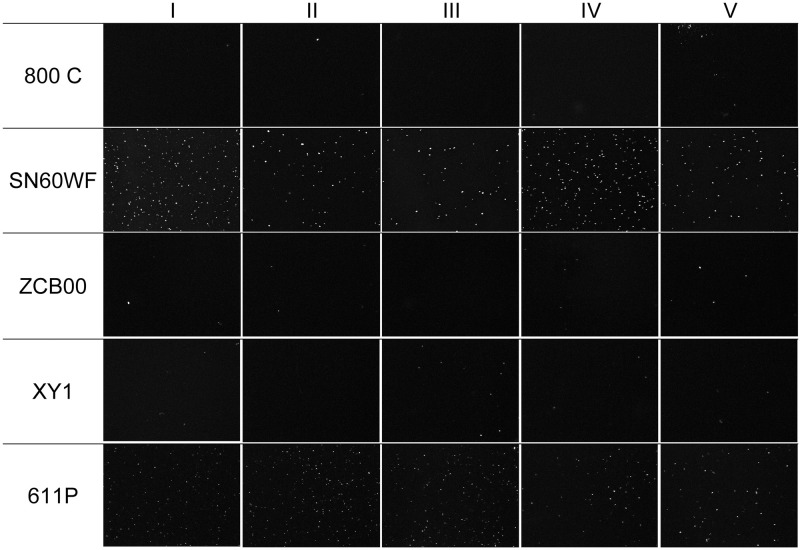
Images in 90x magnification of the central 1200 x 1600 pixels area of all IOLs after glistening induction and image processing. While all the SN60WF and all 611P IOLs developed glistenings, the 800C, ZCB00 and XY1 IOLs show only few glistenings.

There was a low variability in the distribution of glistening number between the five lenses of each group, except for the SN60WF IOLs, where the range between the lenses was wider (Fig 2).

Software image analysis revealed that the number of microvacuoles per square millimetre was highest in the SN60WF and 611P IOLs with 66.0 (±45.5) and 30.7 (±8.4) MVs/mm^2^, respectively. The lowest number of glistenings was obtained averaging the five ZCB00 IOLs, with 0.9 (±0.6) MVs/mm^2^. The 800C IOLs reached statistically significant lower mean glistening numbers compared to the SN60WF and 611P IOLs (P < 0.05), as did the ZCB00 compared to the SN60WF IOLs (P < 0.05) (Table 2). The other inter-group comparisons did not show statistically significant differences.

**Table 2:** Glistening density.

IOL model	(Manufacturer)	Average MV/mm^2^	(±SD)
800 C	(Rayner)	2.0	(±3.6)^a^^,^^b^
SN60WF	(Alcon)	66.0	(±44.5)^a^^,^^c^
ZCB00	(J&J Vision)	0.9	(±0.6)^c^
XY1	(Hoya)	2.7	(±2.4)
611P	(Zeiss)	30.7	(±8.4)^b^

Comparison of the mean microvacuoles per square millimetre (± standard deviation) of the studied intraocular lenses.

IOL intraocular lens; MV/mm^2^ microvacuoles per square millimetre, SD standard deviation;

^a,b,c^ statistically significant differences Mann-Whitney-U-test (Bonferroni corrected).

### Glistening grading

**Fig 3:**
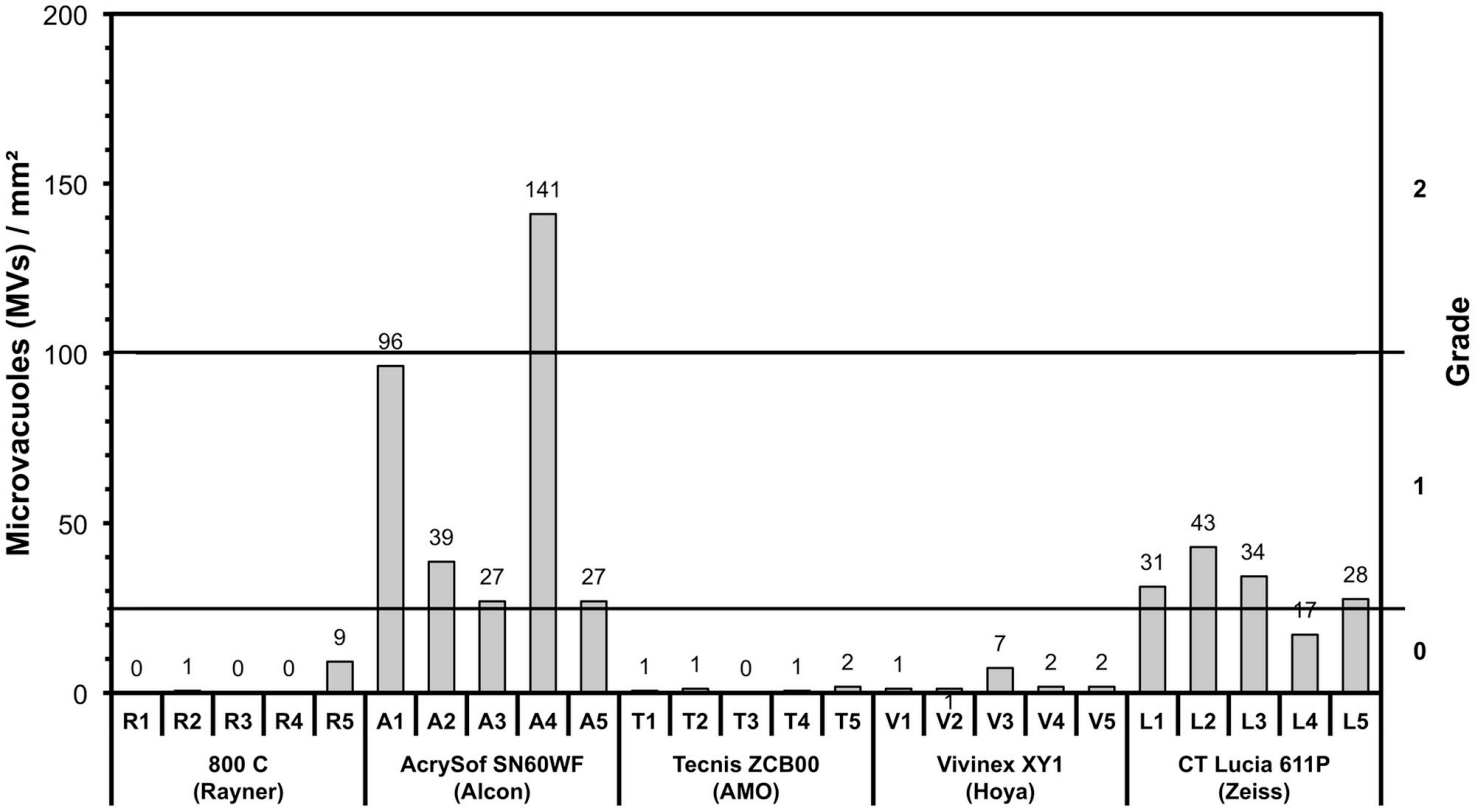
Number of glistenings of all tested IOLs obtained from image analysis after accelerated glistening induction. The secondary y-axis shows the relationship to the grading system. MVs/mm^2^, microvacuoles per square millimetre.

On the glistening grading scale, all ZCB00, 800C and XY1 IOLs were rated as grade 0. Four of five 611P IOLs reached grade 1, one was rated as grade 0. Four of the five SN60WF IOLs were rated grade 1, one reached grade 2 (Fig 3).

## Discussion

The presented study reveals differences in the resistance to glistening formation between acrylic IOLs made of different hydrophobic materials. All are in current clinical use, and some are claimed to be glistening-free. The ZCB00, 800C and XY1 showed mean glistening numbers close to zero, whereas the glistening number was higher in the SN60WF and 611P IOLs (66.0 (±45.5) and 30.7 (±8.4) MVs/mm^2^, respectively. We performed a direct comparison of these IOLs using the same conditions across all samples. We provide the glistening numbers for the 800C IOL: with a mean of 2.0 (±3.6) MVs/mm^2^, it is close to the XY1, which has a mean number of 2.7 (±2.4) MVs/mm^2^. The study revealed that the mean glistening number of the 800C was lower compared to that of the AcrySof SN60WF and the 611P IOLs (P < 0.05, Bonferroni corrected Mann-Whitney-U-test).

Glistenings have been investigated in clinical and laboratory studies for most of the IOL models used in our study, except for the 800C, but this is the first presentation of a direct comparison of these IOLs [2, 11, 15–21]. Clinical data is available for the SN60WF, ZCB00, 611P and XY1, with varying follow-up periods [15–20]. A prospective randomized clinical study from 2002 on 273 patients who received one of 7 different hydrophobic IOLs, including an unknown AcrySof IOL model, found that 40.0 to 67.5% developed glistenings within 2 years [20]. Another clinical study by Colin et al. from 2012 included 111 eyes implanted with AcrySof SN60WF IOLs that were followed for 11 to 80 months. Glistenings occurred in 86.5% of eyes with 40.5% classified as grade 1 and 45.9% as grade 2 [16]. Results of both are consistent with our findings, as we were able to induce glistenings in all of the AcrySof SN60WF IOLs. A prospective randomized, intra-individual clinical trial by Johansson from 2017 compared ZCB00 with SN60WF IOLs. The authors observed a high number of glistenings in most of the SN60WF IOLs, which increased between the 2- and the 3- year visits. The ZCB00 IOLs, on the other hand, only showed a few glistenings without an increase over time [17]. This is also in accordance with our results, which showed a consistently high resistance towards glistening formation for the ZCB00 in vitro compared to the SN60WF. For some modern hydrophobic acrylic IOL materials, clinical studies even suggest a complete absence of glistenings, even 3 to 5 years after surgery [18, 22]. Kahraman et al. compared the SA60AT IOL with the ZCB00 and even after 5 years, none of the eyes with the ZCB00 IOL showed any glistenings [18]. The clinical performance of another IOL from our study, the 611P, was assessed by Borkenstein in 2018. In their study, the authors followed in 96 eyes up to 1 year after surgery and reported an absence of any glistening in all of their cases [15]. This clinical finding seems inconsistent with our results, as the accelerated aging suggests that the 611P IOL is prone to develop glistenings. However, with a mean of 30.7 (±8.4) MVs/mm^2^, the glistening number was rather low. Therefore, the lack of glistenings observed clinically might be explained by the rather short follow-up period of one year, which might not have been enough for the glistenings to develop. Recently, another clinical study assessed glistening formation in the XY1. The authors reported the absence of any glistenings in their cases but follow-up was limited to 3 months after surgery, so long-term data is needed to confirm their findings [19]. However, results from our current study support that a low number of glistenings might be expected in XY1 IOLs.

As manufacturers often claim to have found proprietary ways to produce IOLs with high resistance against material changes like glistenings, apart from clinical investigations, laboratory studies have been conducted to investigate such claims [2, 11, 21]. In 2013, Thomes and Callaghan quantified the results of improvements in the manufacturing process of the AcrySof material. The Authors compared AcrySof SB30AL IOLs manufactured in 2003 with AcrySof SN60WF IOLs made almost ten years later in 2012. The 2012 manufactured AcrySof SN60WF demonstrated a significant reduction in glistening number (39.9 ±35.0 MV/mm^2^) compared to AcrySof SB30AL produced in 2003 (315.7 ±149.4 MV/mm^2^) [2]. In our present study, we found slightly higher values for the AcrySof SN60WF IOLs (produced in 2017) compared to the ones studied by Thomes and Callaghan from 2012, with a mean central glistening number of 66.0 (±45.5) MVs/mm^2^. Nevertheless, numbers were lower than the ones reported for 2003, supporting the argument that the manufacturing process has been improved. Even though improvements in the AcrySof material between 2003 and 2012 led to an increasing resistance to glistening formation, an undesirable amount of glistenings is still present in these lenses [2]. Werner et al. reported on material clarity characteristics in different IOLs including the ZCB00 and C/XY1 IOLs using a similar in vitro method for glistening induction as presented in our study. The authors found a mean glistening number of 2.6 (±2.0) and 4.0 (±2.6) MV/mm^2^ for the two lenses, respectively [21]. Mean glistening numbers in our study for ZCB00 and XY1 were slightly lower with 0.9 (±0.6) and 2.7 (±2.4) MVs/mm^2^, respectively. A former study from our group from 2018 used the same accelerated aging setup and reported glistening numbers for the CT Lucia 601P IOL, a predecessor of the 611P used in the current study, which is made from the same hydrophobic IOL material. The authors reported a mean glistening number of 85 (±86) MV/mm^2^, which is higher compared to the 30.7 (±8.4) MVs/mm^2^ from the current work [11]. One way to explain this difference might be that there was an improvement in the manufacturing process between the 601P and the 611P, which lead to a higher resistance to glistening formation. The Alcon company recently introduced a new material, named Clareon, that is considered to have minimal tendency towards glistening formation. The compounds of the Clareon material are based on the ones of the AcrySof material but phenylethyl methacrylate was replaced with hydroxyethyl methacrylate which increased its equilibrium water content (EWC) to around 1.5% [23]. Glistening number for the Clareon material was reported to be as low as 0.62 (±0.95) MV/mm^2^ in a laboratory study [21]. Nevertheless, the AcrySof material is still sold worldwide and is even used for a recently introduced IOL model with a new extended depth of focus optical design [24].

Many clinical studies on the impact of glistenings did not reveal a strong impact on the visual acuity (VA) or contrast sensitivity (CS) [25]. These findings encouraged the controversy about the relevance of glistenings on optical performance of IOLs [26, 27]. Additionally, in vitro studies have demonstrated that only a large number of glistenings affects the modulation transfer function (MTF) and Strehl ratio [8]. However, other studies suggested that glistenings affect the visual acuity clinically [28, 29]. After further evaluation of this condition, it was found, that glistenings mainly increase intraocular light scattering that does not necessarily translate into clinical symptoms of reduced VA or CS. Straylight parameter (log(s)) was found to be increased in eyes with glistenings grade 2 or higher compared to population-based data of pseudophakic eyes of the same age group [11, 30]. Labuz et al. postulated that straylight increases linearly in dependence of the number of microvacuoles per square millimetre [11]. In this study, glistenings were induced in six different hydrophobic IOL models. Lenses with an elevated number of glistenings were found to have the potential to induce symptoms that could result in difficulties for patients while driving [11].

Hydrophobic acrylic IOL material has certain advantages over other materials: a lower rate of posterior capsule opacification compared to hydrophilic acrylate and PMMA IOLs, and absence of complications associated with hydrophilic acrylate lenses like IOL calcification [31–33]. For these reasons, hydrophobic acrylic IOLs gained popularity and are now widely used despite the known long-term material changes like glistenings and SSNG [7]. As seen in the Clareon material, one approach for manufacturers to reduce the tendency for glistening formation would be to increase the amount of hydrophobic polymer in the composition to increase the IOL material’s hygroscopy (equilibrium water content—EWC) [5, 6]. The IOL’s hygroscopy is usually low in hydrophobic materials: in the AcrySof material the EWC is as low as 0.1–0.5% [34]. There are several more recent hydrophobic materials that incorporate a higher amount acrylate with hydrophilic groups, increasing the material’s EWC [5]. Companies usually do not disclose the exact copolymer composition used for their IOLs. However, the composition of the enVista IOL material by Bausch & Lomb (New York, USA) has been described in the literature [5]. The so called PHS copolymer consists of three acrylic monomers: poly(ethylene glycol) phenyl ether acrylate (40%), hydroxyethyl methacrylate (HEMA, 30%) and styrene (26%), cross-linked by ethylene glycol dimethacrylate (4%). The hydrophilic groups of the HEMA increase the material’s EWC to about 4%. Studies have demonstrated the absence of glistenings in the enVista [5, 35]. Other manufacturers, like PhysIOL (Liège, Belgium) developed hydrophobic IOL materials with an elevated EWC around 5% to reduce the chance of glistening formation [36]. In our study, lenses with a low EWC seem to have a higher tendency towards glistening formation, with EWC of 0.1%–0.5% in the SN60WF and 0.3% in the 611P. The EWC of the other lenses from our study could be higher (Table 1) but the manufacturer does not disclose its exact amount.

In addition to the material properties, the manufacturing process of IOL appears to play an important role in resistance to glistenings. At present, there are mainly two alternative ways of making an IOL, by lathe-cutting or cast-moulding. Some of the lenses from our study are made by cast-moulding (800C, XY1 and SN60WF), some by lathe-cutting (611P and ZCB00). Both methods offer advantages: IOLs made by lathe-cutting possibly retain a more homogenous copolymer distribution within the final IOL compared to the ones formed by cast-moulding where there might be a rearrangement of the polymer distribution [37]. In our study we did not find that one of the methods provided a superior stability regarding glistening formation. The control of polymerization temperature, water content and hydrophilic bonds of the material are other factors considered to be involved in the occurrence of glistening [12]. As some of the manufacturing and polymer characteristics are kept proprietary, the reasons for differences in material stability and long-term clarity still remain unknown. Our study has some limitations: even though we used the same dioptric power (+21D) for all IOLs and we assume the optic size is 6 mm as specified by the manufacturer we cannot exclude a bias due to slight differences in the optic volume due to differences in optic diameter and thickness between each IOL group, differences that arise from the different refractive indexes of the IOL material. We present original measurements made on a lens model (the Rayner 800C) not reported before by an independent laboratory and compare this with a replication of measurements on IOL models that we examined in earlier studies, such as the Acrysof model. (Note, lenses were not reused from previous work, instead for each model we measured new lenses.) The comparison a new lens model with IOL models for which we already have data, establishes our confidence in the reliability of the experimental method for inducing glistenings. Finally, there is the limitation that, as this study was conducted in an in vitro environment, results cannot be translated without qualification to the clinic setting. Long-term clinical studies have to confirm the lower amount of glistenings in IOLs made of advanced hydrophobic materials.

Nevertheless, this laboratory study provides interesting aspects that are based on a direct comparison between IOL models, using the same in vitro accelerated aging procedure across all samples: the ZCB00, 800C and XY1 IOLs are not prone to develop glistenings. Other hydrophobic IOL materials in current clinical use, like the 611P, might have the potential to develop glistenings over time and others, like the SN60WF, develop a higher amount of glistenings. While long-term clinical studies are needed to confirm our in vitro results, manufacturers might consider replacing polymers that show a higher tendency to develop glistenings in vitro with materials that in-vitro studies indicate to be more resistant to this material change.  
